# Pedigree Painter (pepa): a tool for the visualization of genetic inheritance in chromosomal context

**DOI:** 10.1093/bioinformatics/btaf428

**Published:** 2025-07-31

**Authors:** Andrea Pozzi

**Affiliations:** Faculty of Biology, Ludwig-Maximilians-Universität Munich, München, 82152, Germany

## Abstract

**Motivation:**

Data visualization is increasingly important in genomics, enabling researchers to uncover inheritance and recombination patterns across generations. While most existing tools focus on ancestry prediction, they lack functionality for analyzing known ancestries in controlled settings, such as determining parental contributions to offspring genomes. To address this gap, I developed pepa, a lightweight, deterministic, modular tool that visualizes and quantifies genomic inheritance, designed for beginner and advanced users.

**Results:**

pepa is a program for processing VCF files, assigning ancestries to homozygous SNPs, and clustering them into biologically meaningful regions. It generates human-readable comparison tables and visualizes inheritance patterns with chromosome paintings through R. Tested on fission yeast, pepa revealed non-uniform recombination patterns, with chromosomes largely inherited from one parent and seemingly random recombination. Quantitative analyses showed differences in parental contributions at the nucleotide and gene levels, with some offspring inheriting similar percentages from parents. However, the painted chromosomes revealed that even offspring with similar percentages from one parent rarely inherit the same genomic region, highlighting the importance of this tool in drawing biologically meaningful insights. pepa provides an accessible and powerful solution for analyzing genomic inheritance, bridging experimental and computational biology. Its modular design and minimal dependencies allow adaptation to diverse organisms, facilitating intuitive visualization and quantitative insights into recombination dynamics.

## 1 Introduction

The importance of data visualization is rising in biology, as we accumulate an increasing amount of genomic data (Oróstica and Verdugo 2016). Visualizing data is a cornerstone of genomics (Chen *et al.* 2024), enabling researchers to uncover patterns that would otherwise remain unknown. This is particularly important for understanding genome recombination across generations, a topic important across multiple fields, from experimental biology to human evolution (Pritchard *et al.* 2000, Hellenthal *et al.* 2014). Recombination shapes genomes by mixing parental contributions, but the effects observed over a long time (ancestry) often differ from those happening over a short time, e.g. from parents to offspring (pedigree). While the pedigree of an organism is influenced by its ancestry, sometimes the interest focuses on simply understanding which parent-specific parts of the genome can be inherited. One such example is yeast research, where new hybrids are generated multiple times, trying to select specific phenotypes of interest using parental strains with phenotypes of interest.

There are many ancestry prediction and visualization tools, such as ChromPlot (Oróstica and Verdugo 2016) or human-specific commercial tools like Chromosome Painter (AncestryDNA^®^). Most tools focus on ancestry prediction and thus lack versatility in showcasing known ancestries (Chen *et al.* 2024), such as parental contributions to offspring in a controlled setting. This is best exemplified by the software STRUCTURE (Pritchard *et al.* 2000), used by most researchers in population genetics, which includes tools to plot its predicted ancestries. However, similar programs are not helpful when ancestries are known (e.g. known parents), and the goal is to measure the contribution of each parent to an offspring genome. Existing tools like STRUCTURE are designed for population-level inference and stochastic modeling, lacking support for deterministic, parent-specific visualization. ChromPlot allows plotting chromosomal features but lacks per-individual inheritance tracing, and commercial tools like Chromosome Painter are tailored to human data and are not openly customizable.

Many existing tools separate ancestry inference from visualization (Price *et al.* 2006, Hellenthal *et al.* 2014, Oróstica and Verdugo 2016), requiring users to run multiple programs and manually match input and output files between them. This multi-step process can complicate workflows and introduce challenges in ensuring consistency and accuracy when integrating results, particularly due to the risk of human error or misformatted data. Pedigree Painter (pepa) fills this gap by providing a lightweight, Bash-based pipeline that directly quantifies and visualizes per-parent genomic contributions in controlled experimental crosses. It enables users to identify inherited genomic regions or genes from each parent, offering detailed insights into recombination patterns in systems such as yeast hybrid selection or common garden experiments.

## 2 Materials and methods

### 2.1 Tool performance

The performance and scalability of the pipeline were evaluated by testing datasets containing 10, 50, and 100 individuals. All runs were executed on a desktop with an AMD Ryzen 5 9600X processor and 32 GB RAM. The pipeline completed in ∼6.4 s for 10 individuals, and ∼33.9 s for 50 individuals, using peak memory of 185 MB and 347 MB, respectively. For 100 individuals, processing completed in 76.2 s with a peak memory usage of 588 MB. In all tests CPU usage was ∼99%, suggesting good use of available hardware without wasting cycles. Both runtime and memory usage scaled approximately linearly with the number of individuals, demonstrating the efficiency and scalability of the approach. Linear memory scaling is especially advantageous as it ensures predictable resource requirements and prevents sudden spikes in memory consumption, enabling the pipeline to handle increasing genomic complexity without unexpected demands on system resources.

### 2.2 Pipeline design

**Figure 1.**
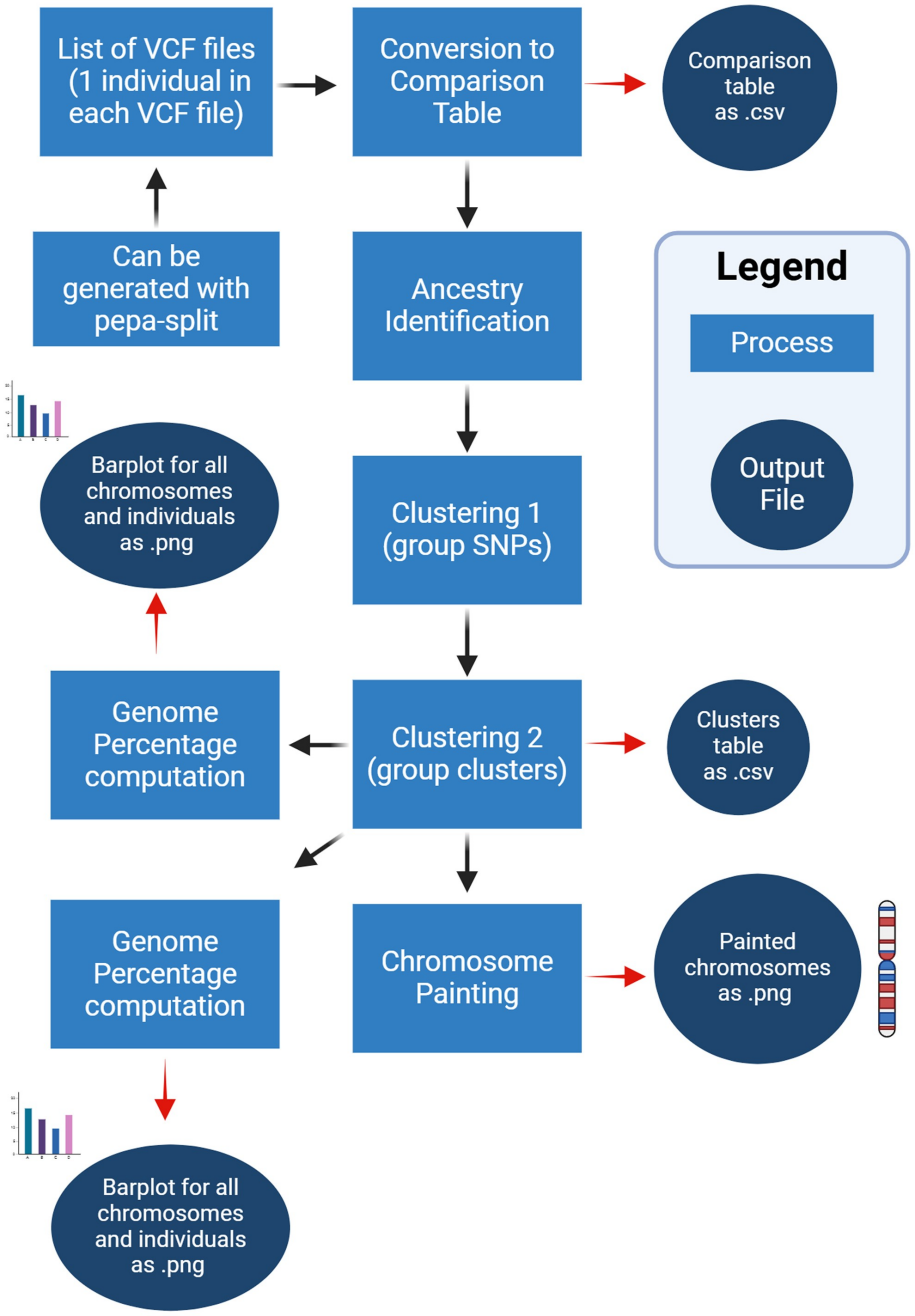
Overview of the pepa workflow. The pepa pipeline begins with a list of VCF files, each containing variant data from a single individual. If a combined VCF is used, the user first applies pepa-split to separate individuals. The VCFs are converted. Created in BioRender. Pozzi, A. (2025). https://BioRender.com/f33x053

*Pepa* is a versatile and accessible tool for visualizing genomic inheritance, recombination patterns, and parental contributions (Fig. 1). Throughout this manuscript, I refer to the short-term parental contribution as *ancestry*. While *pepa* can technically detect ancestry signals at the population level, its primary focus is on pedigrees and controlled crosses. The tool is designed with ease of use and flexibility in mind, enabling both beginner and advanced users to analyze their data effectively. The pipeline is implemented using Bash, Python, and R. Bash serves as the backbone, coordinating the execution of Python and R scripts. Graphics are computed using R, a common language for graphics in biology, with use spanning from transcriptomics (Love *et al.* 2014, Pozzi 2024) to population genetics (Lawrence *et al.* 2013, Oróstica and Verdugo 2016). To make this tool useful to more advanced users, the output tables are saved and optimized for R packages such as ggplot2, allowing users to easily customize their plots with custom colors, shapes and text. The pipeline is lightweight and requires minimal installation. The core tool (*pepa-base***)** only requires Python 3, without extra modules or installation. The extended pipeline, which includes data visualization (*pepa-paint***)**, relies on three common R packages to generate graphics (dplyr, patchwork, ggplot2) (Wickham 2016, R Core Team 2021).

### 2.3 Tool applicability

*Pepa* is designed primarily for haploid or fully homozygous diploid systems, where ancestry assignment is straightforward because each locus unambiguously matches one parental allele. In diploid organisms, heterozygous SNPs (e.g. genotypes 0/1 or 1/0) are excluded to avoid ambiguous assignments, retaining only sites where both alleles match one parent. This approach reduces the number of informative sites in typical diploid datasets but allows reliable detection and visualization of ancestry transitions arising from recombination events such as crossing over. Also, this approach does not require phased genomes. Phasing, which assigns variants to specific parental chromosomes, can be challenging, especially in non-model organisms. By focusing on homozygous SNPs and matching alleles directly to parental references, *pepa* simplifies the process at the cost of excluding heterozygous positions and some resolution. This tradeoff prioritizes accessibility and robustness for a wide range of datasets, including those where phasing is unavailable or unreliable. Nonetheless, *pepa* has not been tested with polyploid organisms, where multiple alleles per locus introduce additional complexity.

In contexts where individuals come from hybrid populations or lack known pedigrees, *pepa* relies on carefully selected reference genomes to represent each ancestral source. This is not the main aim of *pepa*, and SNP selection profoundly influences ancestry assignment accuracy. Therefore, it is recommended to replace the parental genomes with synthetic reference genomes that encapsulate the most representative SNPs of each population or species. Examples of representative SNPs can be those common within groups or linked to key traits (e.g. plumage color or smell). These reference sequences enable the estimation of parental contributions by counting inherited genomic regions or genes assigned to each reference. While this approach has not yet been applied to empirical datasets within *pepa*, future updates plan to include example datasets and workflows illustrating how synthetic references can be leveraged to analyze hybrid genomes such as the ones seen in hybrid zones (Knief *et al.* 2019). This flexibility extends *pepa’*s utility beyond traditional pedigree studies and into broader population genetics applications.

### 2.4 Comparison tables and ancestry identification

The first script of *pepa* takes a list of VCF files, one for each individual under analysis, to generate comparison tables summarizing genetic similarities and differences in a human-readable format. At this stage, all heterozygous SNPs are discarded to ensure that only unambiguous ancestries are considered. This functionality mirrors that of tools like *vcftoolz* (Davis 2019), but with the advantage of requiring no additional installations or dependencies. Each SNP in this table is then assigned an ancestry, from either of the parents. These parents are indicated in the command line, and they can be actual parents (e.g. mother and father), or, when working on hybrid species, they can be two representative genomes from those species. The latter option is valuable when analyzing individuals from a hybrid zone, such as seen with crows in Europe (Knief *et al.* 2019). The table generated is then used to compare SNPs between parents and offspring, retaining only those SNPs that are present in at least one of the parents and discarding all others. For each retained SNP, the script assigns an ancestry label based on which parent the offspring’s allele matches. The analysis is performed row-by-row and sequentially, making the process slower but memory-efficient, allowing it to run on standard desktop machines.

### 2.5 Clustering

*Pepa* includes clustering functionality designed to group SNPs with similar ancestry, addressing the challenge of visualizing millions of individual data points with a computationally efficient approach. The first clustering algorithm sequentially processes SNPs sorted by chromosome and position for each individual, grouping continuous SNPs that share identical ancestry into clusters. When a change in ancestry or chromosome is encountered, the current cluster is finalized only if its length meets or exceeds a user-defined minimum size threshold, specified through the *CLUSTER* parameter. This filtering step is essential to remove biologically insignificant clusters that may arise from erroneous or sporadic SNP calls, such as clusters shorter than 10 nucleotides (nt). The choice of a user-defined threshold recognizes that recombination tract lengths vary widely among species and experimental contexts, so a fixed cutoff would not be appropriate for all datasets. Example outcomes for different cluster sizes (2, 10, 100, 1000, 2000, 5000) are provided in the Test folder on the program’s GitHub page. By allowing users to set this parameter, pepa becomes adaptable to a wide range of organisms, enabling tailored analysis according to the biological characteristics of the species under study. This process continues iteratively until all SNPs are assigned to clusters or discarded, as illustrated in the following pseudocode:

For each individual:

Sort SNPs by chromosome and position

For each SNP:

If ancestry is same as previous SNP:

Extend current cluster

Else:

If cluster length ≥ user-defined CLUSTER threshold:

Output cluster

Start new cluster

Building upon these initial clusters, *pepa* applies a second clustering algorithm that further refines ancestry assignments by merging non-contiguous blocks of identical ancestry separated by small clusters of differing ancestry. These small intervening clusters, typically below a second user-specified size threshold, are often false positive rather than true recombination events. The algorithm scans through the ordered clusters, and when it identifies a small cluster flanked by two larger clusters of the same ancestry, it removes the small cluster and merges the flanking clusters into a single continuous block. For example, if two 10 kb clusters of Parent1 ancestry are separated by a 100 nt cluster assigned to Parent2, this intermediate cluster is discarded, producing a merged 20 kb Parent1 cluster. This approach mirrors biological expectations, as genuine recombination tracts spanning only a few hundred nucleotides are rare in many species. By incorporating user-defined thresholds for minimum cluster sizes and for the size of small intervening clusters to ignore, *pepa* offers flexibility and robustness in ancestry assignment, ensuring that the method can be effectively adapted across diverse taxa and experimental designs.

### 2.6 Chromosome painting

Using R, *pepa-paint* generates visual representations of chromosomes, highlighting regions inherited from each parent. The plotting is based on three widely used R packages (dplyr, ggplot2, and patchwork**),** which together produce per-individual and per-chromosome graphics assembled into a single composite plot. The default implementation is optimized for genomes with a small number of chromosomes (<10) and for medium-sized cohorts (<100 individuals), though the code remains capable of generating interpretable plots for datasets with up to ∼200 individuals and ∼40 chromosomes.

To support custom adaptation, all visualizations are based on standard tabular outputs (dataframes), allowing users with basic R and **ggplot2** knowledge to develop their own scripts for larger or more complex datasets. This design avoids hard-coded assumptions and enables parameter tuning (e.g. cluster size or recombination scale) to better suit different biological systems. Since recombination rates can vary dramatically between clades, this flexibility ensures broader applicability. To assist users in adjusting plots for their data, the Test folder on GitHub includes example visualizations of three yeast chromosomes across 4, 10, 50, and 100 individuals. These were made by analyzing the same yeast individuals multiple times. These figures serve as references to illustrate how visual clarity may change with cohort size, genome structure, and recombination dynamics (e.g. many small clusters versus large blocks).

### 2.7 Quantifying parental contributions

While *pepa* focuses on visualization, it also provides several quantitative outputs. The *pepa-paint* module computes the percentage of the genome inherited from each parent by summing the lengths of ancestry-assigned clusters (-C). Rather than relying on the full genome length, which may be poorly defined in non-model species due to repetitive or invariant regions, *pepa* uses only the informative regions identified through SNP-based clustering. This approach ensures that all mappable and analyzable regions contribute to the quantification, including those ultimately assigned to the “unknown” category.

Importantly, *pepa’*s ancestry assignment is deterministic and does not use statistical inference: it relies on exact SNP matches between parents and offspring. As a result, traditional accuracy metrics like confidence scores or *P*-values do not apply. All the homozygotic SNPs are assigned to one of the three categories (Parent1, Parent2, Unknown), and no errors were ever found using either toy data or real sequencing samples.

*Pepa* also performs gene-level quantification (-G or -A) by intersecting ancestry-assigned regions with annotated gene coordinates provided in a GTF file. This allows users to calculate the number and percentage of genes inherited from each parent. The process is flexible: users can prepare GTF files only with only specific genes (e.g. miRNAs or development-related genes) and *pepa* will use the SNPs to identify from which parent they inherit those genes, generating both a table and a barplot to represent these results. The results are fully dependent on the quality of the annotation file, and errors like fragmented or misannotated genes will directly affect output; this is paramount to use good-quality annotations. Nonetheless, the algorithm of *pepa* deals well with overlapping genes, as a specific ancestry-assigned region inherits that region’s label, even in cases of partial or nested overlaps.

## 3 Results

**Figure 2.**
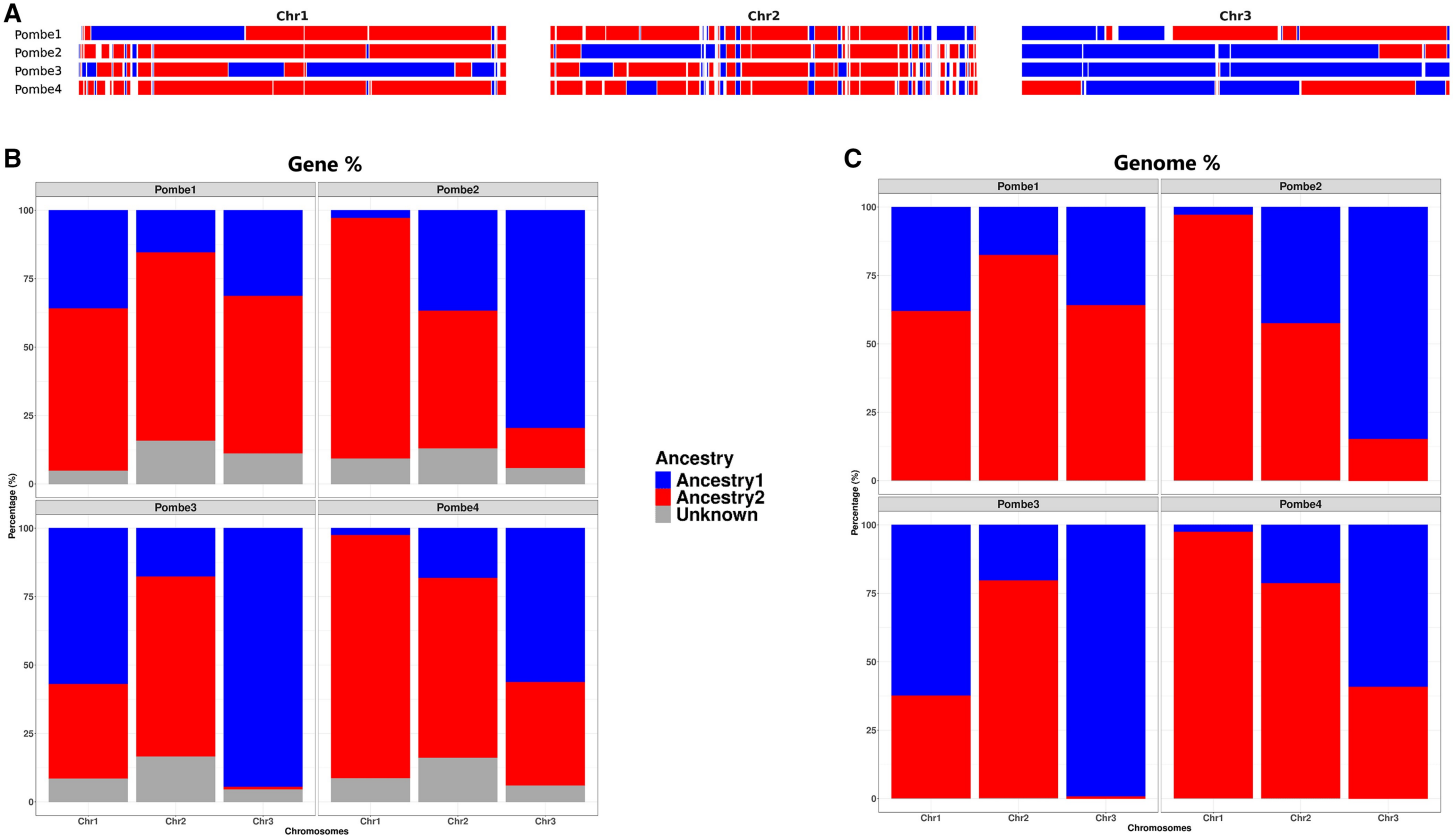
Graphical outputs of pepa analyzing samples from fission yeast. (A) Painted chromosomes for four individuals (Pombe1–Pombe4), showing inheritance from Ancestry1 (blue) and Ancestry2 (red). (B) Barplot showing the percentage of genes inherited from each ancestry across chromosomes (Gene%). Gray regions represent unknown ancestry, such as genes that belong to regions where the ancestry is not assigned. (C) Barplot showing the percentage of the genome inherited from each ancestry across chromosomes (Genome%). Ancestry in all plots is indicated always using the same colors for the same parents.

To highlight the functionality of pepa, I analyzed the offspring of a cross between two strains of fission yeast (Schizosaccaromyces pombe). These strains have been identified before as belonging to two different ancestries, where the compatibility between strains and their phenotypes seems to be affected by the ancestry (Tusso *et al.* 2019). However, while work on identifying ancestry between strains has been performed before, we lack a tool to analyze the pedigree of an experiment, simply tracking from which parent they have inherited portions of their genome. We included here the results from the analyses made with pepa of whole genome sequencing of four offspring (Fig. 2). The painted chromosomes (Fig. 2A) clearly show that each offspring inherited most of Chr1 and Chr2 from the red strain, while Chr3 is mostly from the blue strain. These results align with previous work, where Chr3 has been found to host most of the wtf genes, meiotic drivers that kill offspring if not present in the genome. The painted Chr3 suggests that the blue strain has most of the wtf genes present in the red strain, but the red strain does not have the ones present in the blue one. Thus, the only offspring surviving are the ones inheriting enough Chr3. This inference matches our experience working with these strains, where crosses between these red and blue strains usually have only ∼5% surviving spores, as we obtained only four surviving offspring after culturing 72 individual spores. The inference from the painter chromosome suggests that recombination across regions seems random, but as there are only four samples, I cannot reliably test this effect. Nonetheless, the analysis shows that by using two clustering algorithms, pepa can assign and plot the ancestry of most of the genome across individuals.

The quantitative measurements provided by pepa include the percentage of protein-coding genes (Fig. 2B) and total nucleotides (Fig. 2C) inherited from each parent. For example, Pombe2 shows approximately 15% more of chromosome 2 inherited from the blue parent compared to the other offspring. However, visualization of the painted chromosome reveals that this is largely due to a single, large recombination event in the first half of the chromosome. Similarly, Pombe1 and Pombe3 appear to have higher overall contributions from the blue parent, which might suggest genetic similarity (Table 1). Yet, the chromosome painting reveals that the inherited regions do not overlap substantially, indicating these two strains are genetically distinct despite similar aggregate measures. In contrast, Pombe2 and Pombe4 share more overlapping regions, differing mainly in a large recombination event on chromosome 3, a relatively small chromosome. This example illustrates how combining quantitative summaries with genome-wide visualization enables more accurate and nuanced interpretations of inheritance and recombination patterns.

**Table 1.** Table showing the rounded percentage of ancestry for all the clusters assigned by pepa in each individual and chromosome.

Chr	Individual	Blue (%)	Red (%)	Unknown (%)
Chr1	Pombe1	35.9	59.3	4.8
Chr1	Pombe2	2.9	87.9	9.2
Chr1	Pombe3	57	34.6	8.4
Chr1	Pombe4	2.6	88.9	8.6
Chr2	Pombe1	15.4	68.8	15.7
Chr2	Pombe2	36.8	50.3	12.9
Chr2	Pombe3	17.7	65.8	16.5
Chr2	Pombe4	18.2	65.7	16
Chr3	Pombe1	31.3	57.6	11.1
Chr3	Pombe2	79.7	14.6	5.7
Chr3	Pombe3	94.6	1	4.5
Chr3	Pombe4	56.3	37.9	5.9

## 4 Conclusion

*Pepa* is a lightweight and flexible tool for visualizing genomic inheritance, recombination, and parental contributions in a variety of genetic systems. One of the key insights enabled by *pepa* is that recombination in *S. pombe* hybrids is not uniformly distributed: large chromosome segments are often inherited intact from one parent, with relatively few recombination breakpoints. *Pepa* facilitates the detection and visualization of such patterns, helping to generate biological insights into how inheritance is structured across the genome.

In addition to intuitive visual outputs, *pepa* generates quantitative tables that summarize ancestry proportions genome-wide and per gene, enabling researchers to make objective, reproducible comparisons between individuals. Though formal statistical tests are not yet implemented, future versions of the tool will include statistical assessments of recombination patterns and ancestry proportions, further enhancing its analytical depth. These planned features, along with expanded visualization options, will build upon the current framework to support more rigorous and interpretable analyses. *Pepa* thus provides a practical and extensible foundation for researchers studying inheritance and hybridization in both model and non-model organisms.

## Data Availability

Supplementary data can be found on GitHub https://github.com/Mitopozzi/PePa.

## References

[btaf428-B1] Chen S , Lei C, Zhao X et al AncestryPainter 2.0: visualizing ancestry composition and admixture history graph. Genome Biol Evol 2024;16:evae249. 39545489 10.1093/gbe/evae249 PMC11604083

[btaf428-B2] Davis S. Vcftoolz: a Python package for comparing and evaluating variant call format files. J. Open Source Softw 2019;4:1144.

[btaf428-B3] Hellenthal G, Busby GBJ, Band G et al A genetic atlas of human admixture history. Science 2014;343:747–51. 24531965 10.1126/science.1243518 PMC4209567

[btaf428-B4] Knief U, Bossu CM, Saino N et al Epistatic mutations under divergent selection govern phenotypic variation in the crow hybrid zone. Nat Ecol Evol 2019;3:570–6. 30911146 10.1038/s41559-019-0847-9 PMC6445362

[btaf428-B5] Lawrence M, Huber W, Pagès H et al Software for computing and annotating genomic ranges. PLoS Comput. Biol 2013;9:e1003118. 23950696 10.1371/journal.pcbi.1003118 PMC3738458

[btaf428-B6] Love MI, Huber W, Anders S. Moderated estimation of fold change and dispersion for RNA-seq data with DESeq2. Genome Biol 2014;15:550. 25516281 10.1186/s13059-014-0550-8 PMC4302049

[btaf428-B7] Oróstica KY , Verdugo RA. chromPlot: visualization of genomic data in chromosomal context. Bioinformatics 2016;32:2366–8. 27153580 10.1093/bioinformatics/btw137

[btaf428-B8] Pozzi A. Ancestry affects the transcription of small mitochondrial RNAs in human lymphocytes. Mitochondrion 2024;101907. 38777221 10.1016/j.mito.2024.101907

[btaf428-B9] Price AL, Patterson NJ, Plenge RM et al Principal components analysis corrects for stratification in genome-wide association studies. Nat Genet 2006;38:904–9. 16862161 10.1038/ng1847

[btaf428-B10] Price AL, Patterson NJ, Plenge RM et al Inference of population structure using multilocus genotype data. Genetics 2000;155:945–59. 10835412 10.1093/genetics/155.2.945 PMC1461096

[btaf428-B11] R Core Team. R a Language and Environment for Statistical Computing. Vienna: R Foundation for Statistical Computing. 2021.

[btaf428-B12] Tusso S, Nieuwenhuis BPS, Sedlazeck FJ et al Ancestral admixture is the main determinant of global biodiversity in fission yeast. Mol Biol Evol 2019;36:1975–89. 31225876 10.1093/molbev/msz126 PMC6736153

[btaf428-B13] Wickham H. ggplot2: elegant Graphics for Data Analysis. New York: Springer-Verlag, 2016.

